# Dose and local efficacy analysis of iodine-125 seed implantation therapy for lung tumors

**DOI:** 10.3389/fonc.2025.1589325

**Published:** 2025-06-25

**Authors:** Guohui Cao, Xiaojing Chang, Zeyang Wang, Xiaoli Liu, Ke Xu, Juan Wang, Hongtao Zhang

**Affiliations:** ^1^ Department of Oncology, Hebei General Hospital, Shijiazhuang, China; ^2^ Department of Radiotherapy, the Second Hospital of Hebei Medical University, Shijiazhuang, China

**Keywords:** D90, iodine-125 seed, lung tumor, prognostic factors, tumor size

## Abstract

**Objective:**

To explore the dose and local efficacy of iodine-125 seed implantation therapy for lung tumors.

**Methods:**

The clinical data of 85 patients with lung tumors who underwent iodine-125 seed implantation therapy were retrospectively analyzed. The impact of prescription dose D90 (minimum peripheral dose received by the 90% target volume) on the local treatment effect six months after seed implantation was analyzed, and the critical value for predicting efficacy was determined. Factors affecting the local complete response (CR) rate six months after surgery were also analyzed.

**Results:**

The local control rate at six months after treatment was 89.41% (76/85), and the objective response rate (ORR) was 70.59% (60/85), with a CR rate of 31.76% (27/85). Patients with a post-procedure D90 > 140 Gy had a significantly higher local CR rate. Multivariate analysis revealed that post-procedure D90 and tumor size were independent prognostic factors for achieving CR six months after lung tumor seed implantation.

**Conclusion:**

Iodine-125 seed implantation therapy is effective for lung tumors. Tumor size (*P* = 0.0003) and post-procedure D90 (*P* = 0.0005) were found to be independent prognostic factors for achieving post-procedure CR after lung tumor seed implantation.

## 1 Introduction

The incidence and mortality rates of lung cancer remain the highest worldwide (1). Surgical tumor resection is recognized as the most effective treatment for early- and intermediate-stage lung cancer. However, by the time lung cancer is diagnosed, patients are often already in the intermediate or advanced stages. Owing to their physical condition and other factors, more than 50% of these patients are no longer suitable candidates for radical surgery (2). Currently, the 5-year survival rate of patients with lung cancer is only 19.7% (3). Due to their unique anatomical characteristics, the lungs are the second most common organ for tumor metastasis, with approximately 20%–54% of patients having secondary lung lesions (4, 5). While prior studies have focused on the safety and general local control rates of iodine-125 seed implantation (6–8), consensus on optimal dosimetric parameters—particularly dose-response relationships for tumor eradication—remains elusive. This study systematically defines a quantitative dose threshold (D90 ≥140 Gy) for achieving complete remission in lung tumors. Our findings bridge this critical gap by demonstrating that dose escalation within organ-specific constraints not only significantly improves CR rates but also establishes actionable benchmarks for brachytherapy optimization.

## 2 Data and methods

### 2.1 Study design and patient selection

This retrospective analysis included 85 patients with lung cancer and metastatic lung cancer who were admitted to our hospital between January 2017 and March 2021. The primary disease conditions were detailed in **Table 1**. Among the 85 patients, 64 were male and 21 were female, with an average age of 64.1 (range 41–90) years. Of these, 47 patients received seed implantation combined with systemic treatment, while 38 did not receive systemic treatment. Systemic treatment included chemotherapy, targeted therapy, and immunotherapy, among others. Patients who were pathologically confirmed to have lung cancer through surgery or percutaneous biopsy before treatment and who received standardized comprehensive treatments such as external radiation therapy and chemotherapy, who had confirmed lung tumors before treatment through CT or positron emission tomography (PET)-CT, who were evaluated by two associate chief physicians or higher in the oncology department, who were deemed unsuitable for or refused local treatments such as radiation therapy or surgery, who had a Karnofsky Performance Status score ≥70 points, who had an expected survival time ≥3 months, and who had a normal platelet count and coagulation function were included in the study. This study was approved by the Medical Ethics Committee of our hospital.

**Table 1:** Composition of lung tumors from different primary sources and pathological types.

Primary disease type	Cases	Pathological type	No. of cases
Lung cancer	50	Carcinoma	77
Metastatic lung cancer	35	Sarcoma	8

### 2.2 Iodine-125 seed implantation protocol

The Prowess 3D Version 3.02 close-range treatment planning system (TPS; SSGI, Chico, CA, USA) was used in this study. The equipment used included an 1820-C implantation needle (diameter of 1.22 mm) and a Mick200-TPV20 cm implantation gun (Mick Radio-Nuclear Instruments, Inc., NY, USA). Radioactive iodine-125 seeds, with a γ-ray energy range of 27–35 keV, a radioactive activity of 0.4–0.8 mCi, and a half-life of 59.4 days, were provided by Atomic High Technology Co., Ltd. Preoperative enhanced chest CT scans were routinely performed within one week before iodine-125 seed implantation, and the CT scan data were transferred to the TPS to delineate the 90% dose curve of the target area, including the tumor target volume, and to calculate the number and spatial distribution of the implanted iodine-125 seeds. According to the TPS treatment plan, the prescription dose D90 was set to ≥80 Gy. Timely CT scans were obtained during the implantation process to ensure proper positioning of the seeds according to the TPS. Post-procedure symptomatic treatments, such as electrocardiographic monitoring, oxygen therapy, and hemostasis, were administered. Closed thoracic drainage was performed for patients with significant pneumothorax.

### 2.3 Follow-up and efficacy evaluation

Enhanced chest CT scans were performed at 1, 3, and 6 months post-procedurally to monitor changes in tumor size. The follow-up period was ≥6 months. The local efficacy evaluation of the tumor was based on the Response Evaluation Criteria in Solid Tumors version 1.1 (RECIST 1.1), as follows (9): complete response (CR) referred to the complete disappearance of the tumor, with imaging examinations showing no evidence of the tumor or only linear or thread-like images; partial response (PR) referred to a tumor shrinkage of ≥50%; stable disease (SD) referred to an increase in tumor size of no more than 25% or a shrinkage of less than 50%; and progressive disease (PD) referred to an increase in tumor size of more than 25% or the appearance of new lesions. The objective response rate (ORR) was defined as the proportion of patients achieving either complete response (CR) or partial response (PR) according to RECIST 1.1 criteria (9), with both responses required to be maintained for a minimum duration of 4 weeks.

### 2.4 Statistical analysis

The data were analyzed using SPSS version 25.0 (IBM, Armonk, NY, USA). Independent sample *t* tests were used to analyze the dose–response relationships between the treatment efficacy groups. The optimal D90 threshold of the post-procedure dose was determined by constructing a receiver operating characteristic (ROC) curve. The chi-square test was used to analyze the correlation between various factors and treatment efficacy. Univariate logistic regression analysis was performed for clinical and pathological factors, including variables related to systemic therapies. Binary logistic regression was used for multivariate analysis to evaluate independent prognostic factors while accounting for systemic treatment heterogeneity. A *P* value <0.05 was considered to indicate statistical significance.

## 3 Results

### 3.1 Follow-up

As of September 30, 2021, all 85 patients were followed up for ≥6 months. The local control status of the patients at six months after seed implantation was as follows: 27 (31.76%) achieved CR, 33 (38.82%) achieved PR, 16 (18.82%) showed SD, and 9 (10.59%) showed PD. The CR rate was 31.76% (27/85), the overall response rate was 70.59% (60/85), and the disease control rate was 89.41% (76/85).

### 3.2 Clinical efficacy and analysis

#### 3.2.1 For patients with lung cancer, the post-procedure dose D90 in the CR group was 138.70 ± 19.14 Gy, while in the non-CR group (PR + SD + PD), it was 118.33 ± 28.62 Gy

The difference in post-procedure dose D90 between the CR group and the non-CR group was statistically significant (*t* = 2.34, *P* = 0.024). For patients with metastatic lung cancer, the post-procedure dose D90 in the CR group was 151.33 ± 41.49 Gy, while in the non-CR group (PR + SD + PD), it was 116.76 ± 25.90 Gy. The difference in post-procedure dose D90 between the CR and non-CR groups was statistically significant (*t* = 2.95, *P* = 0.006). There was no statistically significant difference in the post-procedure D90 between the CR group of patients with lung cancer and the CR group of patients with metastatic lung cancer (*t* = 0.99, *P* = 0.335). Similarly, there was no statistically significant difference in the post-procedure D90 between the non-CR group of patients with lung cancer and the non-CR group of patients with metastatic lung cancer (*t* = 0.2, *P* = 0.839) (**Table 2**).

**Table 2:** Comparison of post-procedure D90 dose between the complete response (CR) group and the non-CR group for lung tumors originating from different types of primary cancers.

Primary disease origin	CR group D90 (Gy)	Non-CR group D90 (Gy)	*t*	*P*
Lung cancer	138.70 ± 19.14	118.33 ± 28.62	2.34	0.024
Lung metastases	151.33 ± 41.49	116.76 ± 25.90	2.95	0.006
Lung cancer CR vs metastatic CR			0.99	0.335
Lung cancer non-CR vs metastatic non-CR			0.2	0.839

**Figure 1:**
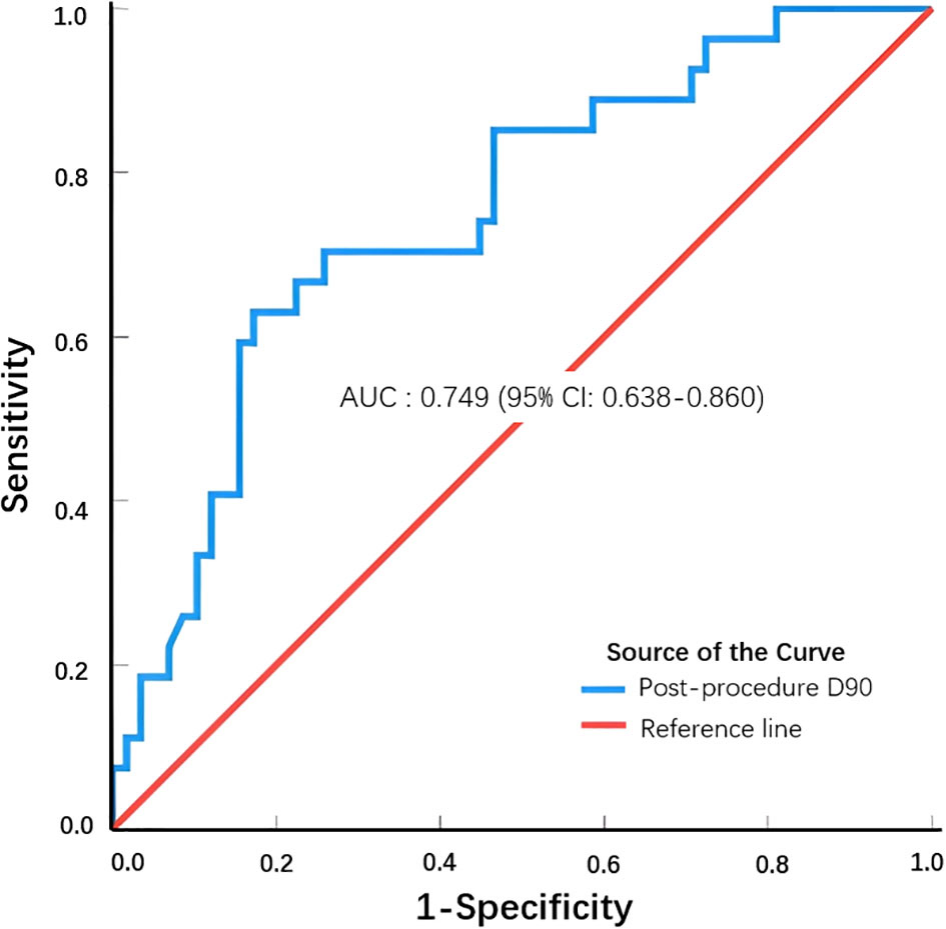
ROC curve to determine the optimal post-procedure D90 threshold to achieve a complete response.

When we combined the CR group patients with lung cancer and metastatic lung cancer and compared them with the non-CR group patients with lung cancer and metastatic lung cancer, we observed a statistically significant difference in D90 between the combined CR and non-CR groups (*t* = 3.94, *P* = 0.00; **Table 3**). The optimal thresholds for post-procedure D90 and the CR rate were obtained by creating an ROC curve, with an area under the curve of 0.749. The maximum Youden index corresponded to a post-procedure D90 of 140.46 Gy (**Figure 1**). When comparing groups based on a post-procedure D90 cutoff of 140 Gy, the CR rates for the D90 ≥140 Gy and D90 <140 Gy groups were 60.71% (17/28) and 17.54% (10/57), respectively, and the difference was statistically significant (*χ^2^* = 16.14, *P* = 0.0001; **Table 4**).

**Table 3:** Comparison of post-procedure D90 dose between the combined complete response (CR) and non-CR groups.

Primary disease origin	Combined CR group D90 (Gy)	Combined non-CR group D90 (Gy)	*t*	*P*
Lung cancer + lung metastases	145.25 ± 33.93	117.77 ± 27.92	3.94	0.000

**Table 4:** Comparison of CR and non-CR patients based on a post-procedure D90 cutoff of 140 Gy.

Post-procedure D90	CR	non-CR	Rate (%)	*χ^2^*	P
<140Gy group	10	47	17.54	16.14	0.0001
≥140Gy group	17	11	60.71		

#### 3.2.2 Analysis of the correlation between post-procedure local control and clinical characteristics

In this study, we categorized general patient information, tumor histopathological classification, the tumor microenvironment, and treatment into seven clinicopathological factors and performed univariate analysis to examine their associations with the post-procedure CR rate six months after seed therapy. The results showed that both tumor size (*χ^2^* = 16.28, *P* = 0.0001) and post-procedure D90 (*χ^2^* = 16.14, *P* = 0.0001) significantly influenced the effectiveness of seed therapy (**Table 5**). However, age, sex, pathological type, preoperative hemoglobin level, and whether combined systemic therapy was administered did not significantly differ. Subgroup analysis demonstrated no significant difference in complete response (CR) rates between patients receiving combined systemic therapy (29.8%, 14/47) and those treated with iodine-125 seed implantation alone (34.2%, 13/38) (*χ^2^ =* 0.19, P=0.663). When factors such as tumor size, post-procedure D90, and whether combined systemic therapy was administered were included in a binary logistic analysis, tumor size (*P* = 0.0003) and post-procedure D90 (*P* = 0.0005) were found to be independent prognostic factors for post-procedure CR.

**Table 5:** Univariate analysis of the post-procedure 6-month complete response rate in 85 patients with seed pulmonary tumors treated with seed therapy.

Characteristic	Cases	CR	Non-CR	Chi-square value	*P*
Sex				1.58	0.208
Male	64	18	46		
Female	21	9	12		
Age (y)					
<65	41	13	28	0	0.991
≥65	44	14	30		
Preoperative hemoglobin (g/L)				0.08	0.775
<120	39	13	26		
≥120	46	14	32		
Pathological type				1.51	0.219
Carcinoma	77	26	51		
Sarcoma	8	1	7		
Tumor size (cm)				16.28	0.0001
<4	42	22	20		
≥4	43	5	38		
Post-procedure D90 (Gy)				16.14	0.0001
<140	57	10	47		
≥140	28	17	11		
systemic therapy				0.19	0.663
Yes	47	14	33		
No	38	13	25		

### 3.3 Complications

Among the patients who underwent seed implantation, 43 had pneumothorax, accounting for 51.4% (43/85) of the total cases. Of these, 12 patients were classified as having small pneumothorax, which resolved spontaneously without symptomatic intervention. For the remaining 31 patients, closed thoracic drainage was performed, and the drainage tube was removed after three days. Bleeding was reported in three patients: Two cases of intrapulmonary bleeding, one of which was accompanied by hemoptysis. Both patients received symptomatic hemostasis, with resolution achieved within 48 hours. One case of subcutaneous hematoma at the implantation site, which resolved spontaneously within two days without intervention. No significant pain, tumor implantation, metastasis, air embolism, or radiation pneumonitis were observed post-procedurally.

## 4 Discussion

Lung cancer has the highest morbidity and mortality rates among malignant tumors in China (10). Due to the lack of early diagnosis, nearly 75% of patients are already in the intermediate and advanced stages when they first seek medical attention, and more than half of these patients, including those with recurrent lung cancer and lung metastasis, miss the optimal window for surgery (11). Studies have shown that metastasis accounts for more than 90% of tumor-related deaths, and 30% to 40% of patients with malignant tumors will develop lung metastasis (12, 13). With the clinical application of the TPS system, iodine-125 radioactive seed implantation, a minimally invasive and effective brachytherapy method, has been widely used for various tumors, such as lung cancer, prostate cancer, and liver cancer (14–17). Numerous studies have shown that seed implantation therapy is highly effective and safe for treating lung cancer and lung metastasis. This article reviews and analyses studies on the treatment of lung tumors with iodine-125 seed implantation, focusing on the relationship between local efficacy and dose.

This study revealed that the local control rate of seed implantation therapy was 89.41% (76/85), the ORR was 70.59% (60/85), and the CR rate was 31.76% (27/85). Wang Z et al. reported a 6-month local control rate of 85.18% and an effective rate of 48.14% for patients with lung cancer. Although the local control rate was consistent with our findings, the effective rate was significantly lower than that in our study (18). Another study by Li et al. showed that with an average D90 value of 132 Gy for seed implantation, the 6-month local control rate for patients with pulmonary metastases was 87.69%, and the effective rate was 78.46%. The local control rate was similar to ours, but the effective rate was greater (19). Li et al. (20) reported that the local control rate of iodine-125 brachytherapy combined with sorafenib for treating liver cancer patients with lung metastasis was 82.4%, which was lower than the rate observed in our study. Vogl et al. (21) compared the local control rates of laser ablation, radiofrequency ablation, and microwave ablation for treating pulmonary metastases from colorectal cancer, reporting rates of 68%, 69.2%, and 88.3%, respectively. The local control rate of seed implantation therapy in our study was comparable to that observed with microwave ablation. While there are numerous studies on the local effective rate and control rate of minimally invasive tumor treatments, few studies have focused on the local complete remission rate. This study analyzed the relationship between the local complete remission rate and dose after seed implantation.

Our analysis suggested that the dose threshold obtained through the ROC curve for achieving CR after seed implantation for lung tumors is 140 Gy. This means that the local CR rate of the tumor significantly increases when a prescribed dose of 140 Gy or more is administered during seed implantation. This dose is equivalent to a biological equivalent dose of 141.91Gy, and when converted into conventional radiotherapy of 2 Gy fractions, it is equivalent to an external exposure dose of 118.26 Gy. The 2021 National Comprehensive Cancer Network (NCCN) guidelines recommend that the biological equivalent dose for stereotactic ablative radiotherapy for non-small cell lung cancer (NSCLC) should be more than 100 Gy for effective local control (22). This study showed that a biologically equivalent dose of 146 Gy or more can achieve better local efficacy. The recommended dose for conventional radiotherapy for NSCLC in the 2021 National Comprehensive Cancer Network (NCCN) guidelines is 60–70 Gy, and dose escalation is associated with better survival (22). From a radiobiological perspective, NSCLC is moderately sensitive to radiation, and studies suggest that a dose of more than 84 Gy is required for more than 50% local control of NSCLC with conventional radiotherapy. However, owing to the limitation of lung tissue tolerance, achieving a dose of more than 60 Gy in conventional radiotherapy is already difficult (23, 24). Radioactive iodine-125 seeds can increase the local tumor absorption dose while reducing radiation doses to adjacent critical organs in the surrounding tissue due to their unique radiobiological characteristic of a high dose gradient. Therefore, extremely high irradiation doses can be administered to the tumor tissue. This study confirmed that the administration of a prescribed dose of 140 Gy or more can significantly improve the CR rate in patients with lung cancer and lung metastases.

Studies have shown that the prognosis for lung cancer tumors larger than 4 cm differs significantly from that for tumors smaller than 4 cm (25). Multiple studies have identified tumor size as a prognostic factor for the efficacy of radiotherapy, which is consistent with the results of this study (26, 27). A possible reason is that as tumors grow, interstitial invasion becomes more obvious, leading to hypoxia within the tumor cells. This hypoxia results in radiation resistance and poor radiotherapy outcomes due to inadequate blood supply. In this study, both univariate and multivariate analyses revealed that post-procedure D90 (with 140 Gy as the cutoff point) and tumor size were independent prognostic factors for achieving a 6-month CR after lung tumor seed implantation. However, this study has limitations such as a small sample size and a lack of long-term follow-up studies; hence, further research and analysis are warranted.

## 5 Conclusions

In conclusion, radioactive seed implantation therapy is a safe and effective method for achieving local control of pulmonary tumors. This study further revealed that administering a prescribed dose of 140 Gy or more can lead to complete remission of local tumors, which is crucial for symptom relief and long-term survival. Although this study conducted detailed analyses of systemic treatment variables (including chemotherapy, targeted therapy, and immunotherapy), the heterogeneity in regimen types and administration timing limited our ability to draw definitive conclusions regarding specific drug effects. Additionally, the small cohort size and absence of long-term follow-up data highlight the need for further research to validate these findings

## Data Availability

The raw data supporting the conclusions of this article will be made available by the authors, without undue reservation.
